# The impact of basic health insurance participation characteristics on the health of mobile populations: the mediating role of health service utilization behavior

**DOI:** 10.3389/fpubh.2024.1243703

**Published:** 2024-02-01

**Authors:** Bo Dong

**Affiliations:** School of Political Science and Public Administration, Wuhan University, Wuhan, China

**Keywords:** health insurance, mobile population, public health services, medical services, China

## Abstract

**Objectives:**

It is a pivotal element of China’s health system reform to improve the health security of health insurance for the mobile population. Achieving this objective is integral to the success of the reform. The aim of this study was to analyze the impact of different enrollment characteristics of basic health insurance on the health of the mobile population and to investigate the mediating role of health service utilization behavior.

**Methods:**

This cross-sectional study included 135,372 migrants who participated in the 2018 China Migrants Dynamic Survey (CMDS). Two indicators were employed in this study to assess the characteristics of the mobile population’s involvement in basic health insurance–namely, whether or not they participated in local health insurance and the type of health insurance in which they participated. The health status of the mobile population was measured using self-assessed health. Health service utilization behavior was divided into public health service utilization and medical service utilization. Multivariate ordered logistic regression was employed to examine the effect of health insurance on the health of the mobile population. Subsequently, the Bootstrap method was applied to analyze the mediating effect of health service utilization behavior in the relationship between health insurance and the health of the mobile population.

**Results:**

Health insurance had a positive impact on health, public health services, and health service utilization among the mobile population. However, enrollment in local health insurance (OR = 1.088, 95% CI = 1.043–1.134) and enrollment in Basic Medical Insurance for Urban Employees (OR = 1.178, 95% CI = 1.090–1.273) were more likely to be associated with higher levels of health and a greater likelihood of receiving health service utilization. The results of the mediating mechanism analysis indicated that health education, health records, family doctor contracting, receiving inpatient services, and being hospitalized locally all played a partially mediating role in the impact of the place of enrollment on health. Regarding the effect of the type of enrollment on health, three types of services–namely, health education, health records, and contracting with a family doctor–played a partially mediating role, while receiving inpatient services and being hospitalized locally did not exhibit a mediating effect. The effect of the type of participation on health is partially mediated.

**Conclusion:**

Based on the impact of the different enrolment characteristics of basic health insurance on the health of the mobile population and the mediating role of health service utilization in this impact, furthermore, improvement of health insurance coverage for the mobile population should focus on improving the accessibility of health services, increasing the level of health insurance coverage, mitigating differences in treatment between the different insurance systems, and simplifying the process of transferring the health insurance relationships.

## Introduction

The concept of the mobile population has developed under China’s household registration system (1), referring to individuals who move and reside outside the designated family registration as stipulated by the household registration system (2, 3). Rapid economic development and accelerated urbanization have led to a redistribution of the population in China (4). An increasing number of people are becoming part of the mobile population due to reasons such as family relocation, reunification, and employment (5, 6). According to the results of the seventh national population census conducted by the Chinese government in 2020, the number of mobile population in China in 2020 will be close to 380 million, an increase of 150 million compared to 2010, representing a growth of nearly 70% (7). With the increase in the scale and frequency of mobility, the health protection of the mobile population has received more and more attention. Previous findings on mobile populations from different countries indicate lower education levels, subpar working and living conditions (8, 9), heightened disease risks (10–12), and a greater likelihood of foregoing necessary health services compared to residents (13–15). Moreover, the majority of public policies and social welfare initiatives in China are formulated and implemented based on household registration (hukou) rather than the actual population residing in a specific region (16). Consequently, many social welfare benefits, including medical insurance, are restricted to urban residents with registered household status, leaving the mobile population unable to fully access or with limited access (17). This results in the mobile population facing heightened health losses and a deteriorated health condition.

Medical insurance is a crucial health protection system that can positively impact the health of the mobile population by reducing the economic barriers to medical services and increasing the accessibility of medical services (18). However, China’s unique medical insurance system leads to differentiated effects on the health of the mobile population based on different enrollment characteristics. Firstly, this is because China’s medical insurance is determined by the location of household registration (18). Under the urban–rural dual system in China, the place where the mobile population participates in medical insurance may not align with their actual residence. Moreover, due to the lower coordination level of China’s medical insurance, the fragmented issues caused by localized management make it challenging for the mobile population to transfer their health insurance relationships. The reimbursement procedures for medical expenses are complex, and the costs are high. Mobile populations often need to spend more time reimbursing medical expenses incurred in locations other than their household registration, and they are required to prepay medical expenses for treatment in other places. Secondly, the differences between various types of medical insurance systems in China also impact the equitable access of healthcare services for the mobile population. China’s healthcare insurance system revolves around basic medical insurance, supplemented by medical assistance and commercial medical insurance, forming a multi-tiered healthcare protection system (19, 20). Among these, basic medical insurance includes rural and urban residents’ basic medical insurance as well as urban employees’ basic medical insurance. These two distinct types of healthcare insurance systems target different insured populations, who not only differ in their occupations but also in the level of healthcare protection they receive. Some studies indicate that compared to rural and urban residents’ basic medical insurance, participating in urban employees’ medical insurance allows the mobile population to access a higher percentage of medical expense reimbursement and a more comprehensive range of healthcare services (21–23). The disparities between these insurance types also affect the healthcare protection level for the health of the mobile population. Moreover, there are differences in the quality of medical services and accessibility between various regions in China. In China, the government plays a dominant role in healthcare services and carries significant fiscal responsibilities in the allocation of medical resources (24). However, due to the implementation of a “graded management and separate funding” fiscal investment system, local governments bear the primary fiscal powers and expenditure responsibilities in the field of healthcare (25). As a result of substantial variations in the fiscal capacities of different regional governments, there are differences in the government’s fiscal contributions to healthcare resources. This leads to variances in the quality of medical services and accessibility between different regions, and these disparities may also impact the health of the mobile population.

The relationship between health insurance and the health of the mobile population can be summarized into three aspects based on relevant research. Firstly, the impact of participating in health insurance on the health of the mobile population has been studied. Research focusing on China’s mobile population suggests that, compared to those without health insurance, participation in health insurance can increase the utilization of medical services and preventive health services among the mobile population (26), leading to an improvement in their overall health (27). Wassink conducted a study on the health insurance coverage and access to medical services for Mexican cross-border returning immigrants, finding a generally low health insurance enrollment rate and poor accessibility to medical services among this group. The study recommends expanding insurance coverage (28). Secondly, the influence of the location of health insurance enrollment on the health of the mobile population remains inconclusive. Some studies analyzing the impact of enrolling in health insurance in the registered residence or current residence on the health of older adult mobile population found no significant differences in health between the two scenarios (1). However, other studies suggest that compared to enrolling in health insurance at the registered residence, enrolling in health insurance at the current residence helps improve the health status of the mobile older adult (29). Meanwhile, research results indicate that mobile populations enrolling in health insurance outside their current residence, due to difficulties in accessing insurance funds, are more likely to forego regular medical service needs (30, 31). Similar conclusions were drawn by Birch and others, who systematically analyzed the achievements and challenges of the Canadian health insurance system. They pointed out that the mobile population faces difficulties in health insurance enrollment and reimbursement processes, reducing their accessibility to medical resources (32). Thirdly, the impact of participating in different types of health insurance on the health of the mobile population shows consistent results in related studies. The research indicates that different types of health insurance systems have varying effects on the health security of the mobile population (33). For example, studies by Zhao and Cai both demonstrate that, compared to participating in rural and urban residents’ health insurance, participating in urban employee health insurance provides higher reimbursement rates and more medical services for the mobile population (20, 21).

On the other hand, some studies have analyzed the mechanisms through which health insurance influences health, summarizing three main pathways. Firstly, health insurance enhances the accessibility of medical services, including regular check-ups, preventive treatment, and high-quality health services, which positively contribute to improving health (34, 35). For instance, Aggarwal’s study on a community-based health insurance project implemented in the Yeshavini region of India found that community health insurance positively promoted the health of insured individuals by increasing the utilization of health services (36). Secondly, health insurance may improve health by influencing individual behavior because participating in health insurance provides access to more preventive healthcare services. Insured individuals may reduce or cease unhealthy behaviors such as drinking or smoking (37, 38). Lastly, health insurance affects health by reducing the cost of obtaining medical services. The reduction in medical expenses implies a decrease in the uncertainty of future medical services and savings in healthcare costs (39). For example, Kim Thuy Nguyen and colleagues, through a survey of 706 households in Dai Dong, Vietnam, analyzed the impact of the Vietnamese health insurance plan on the healthcare expenses and health outcomes of hospitalized and outpatient patients. They found that health insurance, by directly reducing medical expenses and indirectly reducing income losses due to illness, reduced the vulnerability of households facing high healthcare costs (40).

From the aforementioned studies, it is evident that scholars have extensively examined the relationship between medical insurance and the health of the mobile population. Nevertheless, there is still room for further expansion in this area. Firstly, the impact of whether to participate in health insurance at the place of residence on the health of the mobile population is controversial, and relevant studies have not reached a consistent conclusion. Secondly, in terms of research content analysis, existing studies primarily focus on the impact of health insurance on the self-perceived health of the mobile population, with insufficient research on the utilization of health services by the mobile population. Finally, in terms of the influence pathway, current studies mainly analyze the impact pathway of whether or not to participate in health insurance. However, the mechanism through which the characteristics of mobile population participation and the type of insurance affect their health remains unclear. Therefore, further exploration is needed to understand the pathway through which health insurance influences the health of the mobile population. Based on the above analysis and considering that China has already achieved the goal of universal coverage of basic health insurance (29), this paper focuses on studying the characteristics of the mobile population’s participation in health insurance, including whether to enroll locally and the type of enrollment’s impact on the health of the mobile population. Additionally, the study further analyzes the mediating effect of health service utilization behavior in this context. The goal is to improve health insurance policies for the mobile population and enhance their overall health, providing valuable insights for policymaking and raising the health standards of the mobile population.

## Materials and methods

### Study design and data sources

The China Migrants Dynamic Survey (CMDS) is a nationwide large-scale cross-sectional questionnaire survey conducted by the National Health Commission of the People’s Republic of China to monitor the dynamics of the domestic mobile population (2). This nationally representative survey commenced in 2009 and has been conducted annually since. The data utilized in this study is derived from the 2018 nationwide survey on the dynamic monitoring of the mobile population.

The CMDS is acknowledged for its representativeness and minimal sampling error (41). This survey employs a multi-stage stratified probability proportionate to size (PPS) cluster sampling strategy for sample selection (42). In the first stage, townships (towns, streets) are selected using the PPS method. In the second stage, villages (residential committees) within the chosen townships (towns, streets) are sampled using the PPS method. In the third stage, individuals are selected for the survey within the chosen villages (residential committees). Rigorous measures have been implemented to ensure data quality, including scientifically designed questionnaires, training for surveyors, survey supervisor verification of questionnaires, and quality checks through telephone follow-ups.

The CMDS encompasses a rich set of variables (43) and spans across 32 provincial-level administrative regions in China (31 provinces, autonomous regions, municipalities, and the Xinjiang Production and Construction Corp). The survey collected a total of 152,000 samples from the mobile population. The target population for this survey includes individuals aged 15 or above who have resided outside their registered residence (urban or rural) for more than 1 month. Consequently, this study defines the mobile population as those residing in their current location for 1 month or more without local residence registration. The content of the CMDS includes not only demographic and socio-economic characteristics of respondents and their family members but also their health status and utilization of public health services and medical services. For this study, samples with missing values in important variables, extreme values, and those participating in two or more health insurance programs were excluded. The final analytical sample size is 135,372, accounting for 89.06% of the total survey samples.

### Variables

#### Dependent variable

In this study, self-rated health is used to assess the health status of the mobile population, a commonly used indicator in previous research (20). Respondents were asked about their health status, with four possible responses: unable to take care of oneself, unhealthy but able to take care of oneself, basically healthy, and healthy. Therefore, this study categorizes these four results into three situations: combining unable to take care of oneself and unhealthy but able to take care of oneself as unhealthy, and considering basically healthy and healthy as separate categories. These three situations are assigned values of 1, 2, and 3, representing the respective outcomes. Self-rated health is thus an ordered variable, with higher values indicating better health conditions.

#### Independent variable

The independent variables in this study focus on the insurance characteristics of the mobile population, which, as mentioned earlier, can be divided into three categories: whether to participate in health insurance, the location of health insurance participation (i.e., whether to participate in health insurance at the place of residence), and the type of health insurance participation. Considering China’s extensive medical insurance coverage and the achievement of universal coverage of basic health insurance, the study defines insurance characteristics as two scenarios. Firstly, a binary variable is established to indicate whether the mobile population participates in health insurance at the place of residence, where respondents are asked if they have enrolled in health insurance at their place of residence (yes = 1, no = 2). Secondly, the type of health insurance participation is categorized into two groups: Basic Medical Insurance for Urban Employees (BMISUE) and Basic Medical Insurance for Urban and Rural Residents (BMISURR), with values of 1 and 2, respectively.

#### Mediating variable

Mediating variables analyze the pathway through which health insurance affects the health of the mobile population. In selecting mediating variables, this study, based on existing literature (37, 38) and data availability, chooses the healthcare service utilization behavior of the mobile population as the mediating variable. The study divides the healthcare service utilization behavior into public health service utilization behavior and medical service utilization behavior. Public health service utilization behavior includes whether individuals received health education, measured by the question “In the past year, have you received health education on any of the following aspects in your current place of residence or workplace?” The education includes occupational disease prevention and control, prevention and control of chronic diseases, among seven others. If an individual received education on one or more of these aspects, it is assigned a value of 1; otherwise, it is assigned a value of 2. Other variables include whether individuals have established health records locally (yes = 1, no = 2) and whether they have signed contracts with local family doctors (yes = 1, no = 2). Medical service utilization behavior mainly refers to the hospitalization service utilization behavior of the mobile population, including whether they were hospitalized (yes = 1, no = 2) and whether they were hospitalized at their place of residence (yes = 1, no = 2). Due to the 2018 CMDS survey not including interviews on the outpatient service utilization behavior of the mobile population, this study only analyzes the mediating effect of the hospitalization service utilization behavior between health insurance and health.

#### Control variables

Based on the Andersen Healthcare Service Utilization Model (39, 42) and relevant existing research, combined with data availability, the study selects control variables to adjust for confounding effects. These variables fall into three categories: predisposing characteristics, enabling resources, and contextual characteristics. Predisposing characteristics include gender, age, marital status, and education level (44). As economic, social environments, and health insurance policies vary across provinces (45), the study also controls for the province of residence according to related literature (46). Enabling resources comprise family income level, employment status, and hukou type (43). Contextual characteristics include the reasons for migration and the scope of migration (47). Specific settings based on the selected data conditions are as follows: gender is a dummy variable with males coded as 1 and females coded as 2; age is calculated as the difference between the interview year-month and birth year-month; marital status is a dummy variable where individuals who are initially married, remarried, or cohabiting are coded as 1, and those unmarried, divorced, or widowed are coded as 2; education level is a five-level variable based on individual education: never attended school = 1, primary school = 2, middle school = 3, high school = 4, and college or above = 5; family income level is transformed into rankings within each province (<percentile 20, percentile 20–39, percentile 40–59, percentile 60–79, and ≥ percentile 80) for data analysis; employment status is a dummy variable, with employed coded as 1 and unemployed coded as 2; hukou type is a dummy variable, with urban coded as 1 and rural coded as 2; reasons for migration are coded as follows: work-related migration = 1, other reasons = 2, family-related migration = 3; the scope of migration is coded as follows: Intercity = 1, Interprovince = 2, Intercounty = 3.

**Figure 1:**
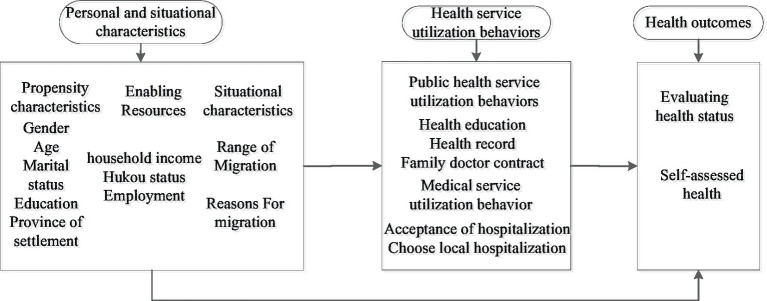
Theoretical framework of mobile population health service utilization behavior based on Anderson model.

Integrating these analyses, our study develops a theoretical framework that examines the interplay among individual characteristics, health outcomes, and health service utilization behaviors within mobile populations. Additionally, it explores how the location and type of health insurance enrollment impact the health of mobile populations through their health service utilization behaviors (Figure 1).

### Statistical method

#### Ordered multicategorical logistic regression model

In this study, the dependent variable, the health of the mobile population, is an ordered variable with values ranging from 1 to 4. Therefore, we employed an ordered logistic regression model to analyze the impact of medical insurance on the health of the mobile population. In the test for the applicability of the model, variables were included in a multivariate ordered logistic regression analysis. The resulting Logit connection function scale models for the location of insurance enrollment and the type of insurance enrollment showed corresponding Sig values of 0.000, which were significant at the 1% significance level, indicating the significant fitting of the regression models. The goodness-of-fit tests for the location of insurance enrollment and type of insurance enrollment regression models using the Pearson and Deviance methods yielded *p*-values of 1.000, indicating that the model adequately fits the data. Simultaneously, in the parallelism test, both p-values were greater than 0.05, suggesting that the location parameters are the same across corresponding categories, meeting the conditions for using the multivariate ordered logistic regression model. The basic form of this model is as follows:


$$\ln\left(\frac{P\left(Y\leq j\right)}{1-P\left(Y\leq j\right)}\right)=\beta_{0}+\beta_{1}X+\beta_{2m}C_{m}+\varepsilon$$

Where Y denotes the health self-assessment status of the mobile population, $\beta_{0}$ is the intercept term, X denotes the location or type of health insurance for the mobile population, $\beta_{1}$ denotes the coefficient of the effect of the location or type of health insurance on health self-assessment, $C_{m}$ denotes other control variables, $\beta_{2m}$ denotes the coefficient of the control variables, ε is the error term.

#### Binary logistic regression model

In this study, given that both the utilization of public health services and hospitalization services among the mobile population is represented as dummy binary variables, we employed a binary logistic regression model to examine the influence of health insurance on health service utilization within this population. After conducting the Hosmer-Lemeshow (HL) goodness-of-fit test, the regression models for the utilization of health services among the mobile population all passed the HL test (*p* > 0.05), indicating that the model fit is good. The basic form of the model is as follows:


$$\log\mathrm{it}\frac{P_{i}}{1-P_{i}}=\beta_{0}+\beta_{1}\mathrm{Insuranc}e_{i}+\beta_{2}\mathrm{Contro}l_{i}+\varepsilon_{i}$$

Here, $P_{i}$ denotes the probability of receiving health care or medical services among the mobile population, $\beta_{0}$ is the intercept term, denotes the participation of the mobile population in health insurance, $\mathrm{Insuranc}e_{i}$ including the location of participation or type of health insurance, $\beta_{1}$ denotes the coefficient of the effect of the location or type of participation on health behavior, $\mathrm{Contro}l_{i}$ denotes other control variables, $\beta_{2}$ denotes the coefficient of the control variables, $\varepsilon_{i}$ is the error term.

#### Bootstrap mediated effects model

According to existing research literature (37, 38), the utilization of health services by insured individuals may play a mediating role between health insurance and well-being. Therefore, to analyze the underlying mechanisms and pathways through which health insurance influences the health of mobile populations, this study employs the Bootstrap method to examine the mediating effects of health service utilization behavior in the impact of health insurance on the health of mobile populations. This choice is based on the significant advantages of the Bootstrap method compared to other mediation analysis techniques, such as Sobel tests and the product of coefficients method (48). First, this method allows for a direct significance test of the coefficients of the mediating effect, without assuming the existence of a significant direct effect. Second, it enables mediation analysis with different levels of moderating variables based on the same model, thereby enhancing testing effectiveness and avoiding data omission issues. In this study, the dependent variable is the self-perceived health status of mobile populations, which is an ordinal categorical variable. The mediating variable is the health service utilization behavior of mobile populations, which is a categorical variable. Therefore, this paper adopts the mediation testing method proposed by Iacobucci in 2012 (49) and utilizes the Bootstrap program in SPSS 23.0, with a sample size set at 5000 and a confidence level of 95% for conducting the mediation effects test. The specific testing model is as follows:

Firstly, establish the following three regression equations. Since the dependent variable in this study is an ordinal variable, the Logit regression model was chosen


$$Y=b_{01}+cX+e_{1}$$

M=b02+aX+e2

$$Y=b_{03}+c^{\prime }X+bM+e_{3}$$

Where X is the independent variable, Y is the dependent variable, M denotes the mediating variable, c , a , $c^{\prime }$ , b are the coefficients to be estimated, where regression coefficient c is the effect of the independent variable X on the dependent variable Y , regression coefficient a is the effect of the independent variable X on the mediating variable M , regression coefficient b is the effect of M on Y after controlling for the effect of X , coefficient $c^{\prime }$ is the effect of X on Y after controlling for the effect of M . $e_{1}$ , $e_{2}$ , $e_{3}$ and is the random error term.

Next, utilizing the coefficients obtained from the above regression models, calculate the following values:

$z_{a}=\hat{a}/{\hat{s}}_{a}$ , $z_{b}=\hat{a}/{\hat{s}}_{b}$ , $z_{a\cdot b}=z_{a}\cdot z_{b}$ , ${\hat{\sigma }}_{z_{\mathrm{ab}}}=\sqrt{{z^{2}}_{a}+{z^{2}}_{b}+1}$ . Finally, $z_{\mathrm{Mediatiaon}}$ is calculated and then the significance of the mediating effect is tested based on its belonging to the normal distribution at the significance level of 0.05, if $z_{\mathrm{Mediatiaon}}>$ 1.96, then the mediating effect is significant. $z_{\mathrm{Mediatiaon}}$ is calculated as follows.


$$z_{\mathrm{Mediatiaon}}=\frac{z_{a\cdot b}}{{\hat{\sigma }}_{z_{\mathrm{ab}}}}=\frac{z_{a}\cdot z_{b}}{\sqrt{{z^{2}}_{a}+{z^{2}}_{b}+1}}$$

## Results

### Characteristics of respondents

Table 1 presents the basic characteristics of the mobile population. The results indicate that 28.56% of the mobile population participates in local health insurance, while 71.44% do not. Regarding the type of health insurance, the majority of the mobile population (76.9%) opts for rural and urban resident medical insurance, with only 23.1% choosing urban employee medical insurance. In terms of public health service utilization, the highest proportion of the mobile population receives health education services (81.78%). Simultaneously, among those participating in local health insurance, the proportion receiving this service (85.92%) is significantly higher than that of those not participating locally (80.13%). For the other two preventive healthcare behaviors, only 33.19% of the mobile population establishes health records locally, and 14.55% sign contracts with local family doctors. Among the mobile population participating in local health insurance, this proportion is only 16.85%. In terms of healthcare service utilization behaviors, only 28.9% of the mobile population chooses hospitalization. Concerning the choice of hospitalization location, 72.98% of the mobile population opts for local hospitals. In other characteristics, the education level of the mobile population is relatively low, with 80.71% having a high school education or below. Over 68% of the mobile population belongs to agricultural households, and 48.75% are inter-province migrants.

**Table 1:** Characteristics of respondents with and without local health insurance.

Variable	Definition	Sample size		Number of respondents				*p* Value
		*n*	%	With local insurance		Without local insurance		
				*n*	%	*n*	%	
Health status	Unhealthy = 1	2,918	2.16	660	1.71	2,258	2.53	<0.001
	Basically Healthy = 2	15,123	11.17	3,685	9.53	11,438	11.83	
	Healthy = 3	117,331	86.67	34,311	88.76	83,020	85.84	
Type of health insurance	BMIUE = 1	30,966	23.10	11,982	31.11	18,984	19.85	<0.001
	BMISURR = 2	103,166	76.90	26,527	68.89	76,629	80.15	
Health education	Yes = 1	110,708	81.78	33,213	85.92	77,495	80.13	<0.001
	No = 2	24,664	18.22	5,443	14.08	19,221	19.87	
Establishing a health record	Yes = 1	36,069	33.19	12,651	38.89	23,418	30.76	<0.001
	No = 2	72,600	66.81	19,878	61.11	52,722	69.24	
Family doctor contract	Yes = 1	15,930	14.55	5,626	16.85	10,304	13.40	<0.001
	No = 2	94,345	85.55	27,754	83.15	66,591	86.60	
Acceptance of hospitalization	Yes = 1	4,423	28.90	1934	31.11	3,029	27.99	0.021
	No = 2	10,879	71.10	3,087	68.89	7,792	72.01	
Choose local hospitalization	Yes = 1	3,228	72.98	1,201	86.15	2027	66.92	<0.001
	No = 2	1,195	27.02	193	13.85	1,002	33.08	
Gender	Male = 1	69,526	51.36	19,762	51.12	49,764	51.55	0.070
	Female = 2	65,846	48.68	18,894	48.88	46,952	48.45	
Age	15–30 = 1	41,467	30.63	13,156	34.03	28,311	29.27	<0.001
	31–45 = 2	61,161	45.18	19,233	49.75	41,928	43.35	
	46–60 = 3	27,298	20.17	5,466	14.14	21,832	22.57	
	61 + =4	5,446	4.02	802	2.07	4,644	4.80	
Education	Illiterate = 1	3,400	2.51	463	1.20	2,937	3.03	<0.001
	Primary school = 2	18,551	13.70	2,806	7.26	15,745	16.28	
	Junior middle school = 3	57,428	42.42	10,785	27.90	46,443	48.23	
	Senior middle school = 4	29,877	22.07	8,788	22.73	21,089	21.81	
	University/college = 5	26,116	19.29	15,814	40.91	1.302	10.65	
Marriage Status	Married = 1	110,845	81.88	30,621	79.21	80,224	82.95	<0.001
	Unmarried = 2	24,527	18.12	8,306	20.79	16,491	17.05	
Employment	Employed = 1	113,112	83.56	34,654	89.64	78,458	81.12	<0.001
	Unemployed = 2	22,260	16.44	4,002	10.36	18,258	18.88	
Hukou status	Urban Account = 1	42,651	31.51	17,754	45.93	24,897	25.74	<0.001
	Rural household registration = 2	92,721	68.49	20,903	54.07	71,818	74.26	
Range of migration	Intercity = 1	45,501	33.61	14,184	36.69	31,317	32.38	<0.001
	Interprovince = 2	65,982	48.75	19,356	50.07	46,626	48.21	
	Intercounty = 3	23,899	17.65	5,117	13.24	18,772	19.41	
Reasons for migration	Work = 1	114,497	84.58	33,625	86.98	80,872	83.62	<0.001
	Others = 2	2,330	1.72	492	1.27	1838	1.90	
	Family = 3	18,545	13.70	4,540	11.74	14,005	14.48	
Household income ranking	Lowest (<percentile 20) = 1	29,799	22.01	5,869	15.18	23,930	24.74	<0.001
	Lower (percentile 20–39) = 2	27,384	20.23	6,662	17.23	20,722	21.43	
	Middle (percentile 40–59) = 3	27,276	20.15	7,489	19.37	19,787	20.46	
	Higher (percentile 60–79) = 4	26,436	19.53	8,865	22.93	17,571	18.17	
	Highest (≥percentile 80) = 5	24,477	18.08	9,772	25.28	14,705	15.20	
Total		135,572	100	38,719	28.56	96,858	71.44	

*P* value in the table were obtained by *χ*^2^ test; BMISURR, Basic Medical Insurance System for Urban and Rural Residents; BMIUE, Basic Medical Insurance for Urban Employees.

### The impact of medical insurance enrollment characteristics on the health of the mobile population

The results of the impact of basic health insurance enrollment characteristics on the health of the mobile population are shown in Tables 2, 3. Table 2 presents the results of ordered logistic regression on the impact of the location of health insurance enrollment on the health of the mobile population, while Table 3 displays the results of the impact of the type of health insurance on the health of the mobile population. From the regression results in Table 2, it can be observed that, compared to those not locally enrolled in health insurance, those locally enrolled are more likely to have a better health status (OR = 1.088, 95% CI = 1.043–1.134). The regression results in Table 3 reveal that different types of health insurance enrollment have differentiated impacts on the health of the mobile population. Compared to rural and urban resident medical insurance, participants in urban employee medical insurance have a greater likelihood of having a higher health status (OR = 1.178, 95% CI = 1.090–1.273).

**Table 2:** Ordered logistic regression results of the location of health insurance participation affecting the health of the mobile population.

Variable		OR	95%CI	*p* Value
Health = 1		0.154	0.137–0.173	<0.001
Health = 2		1.521	1.359–1.702	<0.001
Enrolment with a local health insurance	No (reference)			
	Yes	1.088	1.043–1.134	<0.001
Gender	Female (reference)			
	Male	1.010	0.974–1.048	0.578
Age	61-(reference)			
	15–30	10.084	9.317–10.915	<0.001
	31–45	5.013	4.666–5.386	<0.001
	46–60	2.203	2.053–2.364	<0.001
Education	University/college (reference)			
	Illiterate	0.442	0.401–0.487	<0.001
	Primary school	0.644	0.601–0.691	<0.001
	Junior middle school	0.902	0.849–0.958	0.001
	Senior middle school	0.960	0.901–1.024	0.215
Marriage status	Unmarried (reference)			
	Married	1.211	1.147–1.277	<0.001
Employment	Unemployed (reference)			
	Employed	2.301	2.192–2.415	<0.001
Hukou status	Rural household registration(reference)			
	Urban Account	1.036	0.996–1.078	0.078
Range of migration	Countering (reference)			
	Intercity	1.103	1.052–1.157	<0.001
	Inter province	1.251	1.196–1.310	<0.001
Reasons for migration	Family (reference)			
	Work	1.077	1.020–1.137	0.007
	Others	0.742	0.671–0.822	<0.001
Household income ranking	Highest(≥percentile 80) (reference)			
	Lowest (<percentile 20)	0.697	0.657–0.741	<0.001
	Lower (percentile 20–39)	0.844	0.793–0.898	<0.001
	Middle (percentile 40–59)	0.924	0.868–0.983	0.013
	Higher (percentile 60–79)	0.993	0.932–1.058	0.831
Pseudo *R*^2^		0.114		
*N*		135,372		
Province of settlement		Control		

Due to the covariance between the variables of location of participation and type of participation, we did not include the type of participation in our analysis of the effect of location of participation, the same below.

**Table 3:** Ordered logistic regression results of the type of medical insurance affecting the health of the mobile population.

Variable		OR	95%CI	*p* Value
Health = 1		0.207	0.163—0.264	<0.001
Health = 2		1.910	1.506—2.423	<0.001
Type of Health Insurance	BMISURR (reference)			
	BMIUE	1.178	1.090—1.273	<0.001
Gender	Female (reference)			
	Male	0.976	0.909—1.048	0.503
Age	61-(reference)			
	15–30	11.851	9.870—14.230	<0.001
	31–45	6.645	5.604—7.880	<0.001
	46–60	2.776	2.345—3.287	<0.001
Education	University/college (reference)			
	Illiterate	0.307	0.247—0.381	<0.001
	Primary school	0.573	0.502—0.654	<0.001
	Junior middle school	0.919	0.830—1.017	0.101
	Senior middle school	0.979	0.884—1.084	0.684
Marriage status	Unmarried (reference)			
	Married	1.137	1.026—1.260	0.014
Employment	Unemployed (reference)			
	Employed	2.363	2.137—2.612	<0.001
Hukou status	Rural household registration (reference)			
	Urban Account	1.133	1.053—1.218	0.001
Range of migration	Intercounty (reference)			
	Intercity	1.109	1.001—1.228	0.047
	Interprovince	1.242	1.225—1.371	<0.001
Reasons for migration	Family = 1 (reference)			
	Work = 2	0.941	0.845—1.048	0.269
	Others = 3	0.800	0.627—1.019	0.070
Household income ranking	Highest(≥percentile 80) (reference)			
	Lowest (<percentile 20)	0.687	0.611—0.771	<0.001
	Lower (percentile 20–39)	0.875	0.779—0.982	0.023
	Middle (percentile 40–59)	0.993	0.886—1.114	0.910
	Higher (percentile 60–79)	1.041	0.931—1.164	0.478
Pseudo *R*^2^		0.158		
*N*		38,509		
Province of settlement		Control		

Due to the covariance between the variables of place of enrollment and type of enrollment, we did not include place of enrollment in our analysis of the effect of type of enrollment, the same below. BMISURR, Basic Medical Insurance System for Urban and Rural Residents; BMIUE, Basic Medical Insurance for Urban Employees.

### The impact of health insurance enrollment characteristics on health service utilization behavior of mobile population

The influence of health insurance enrollment location and type on the health service utilization behaviors of mobile populations is depicted in Tables 4, 5. The regression results in Table 4 reveal that enrolling in health insurance locally increases the likelihood of accessing public health services and medical care. Specifically, individuals with local health insurance are more likely to avail themselves of services such as health education (OR = 1.336, 95% CI = 1.290–1.384), health records (OR = 1.505, 95% CI = 1.460–1.550), and family doctor sign-up services (OR = 1.445, 95% CI = 1.390–1.502) in public health service utilization behaviors. Similar conclusions are supported in the healthcare service utilization behaviors of mobile populations, indicating that individuals with local health insurance are more likely to obtain inpatient services (OR = 1.286, 95% CI = 1.179–1.401) and receive inpatient services locally (OR = 3.118, 95% CI = 2.602–3.735).

**Table 4:** Binary logistic regression results of enrollment location affecting health service utilization behavior of mobile population.

Variable			Public health service utilization behavior			Medical service utilization behavior	
			Health education	Establishing a health record	Family doctor contract	Acceptance of hospitalization	Choose local hospitalization
Enrolment with a local health insurance	No (reference)						
	Yes	OR	1.336	1.505	1.445	1.286	3.118
		95%CI	1.290–1.384	1.460–1.550	1.390–1.502	1.179–1.401	2.602–3.735
		*p* Value	<0.001	<0.001	<0.001	<0.001	<0.001
Gender	Female (reference)						
	Male	OR	0.912	0.872	0.914	0.729	0.987
		95%CI	0.885–0.940	0.849–0.897	0.882–0.948	0.672–0.791	0.839–1.162
		*p* Value	<0.001	<0.001	<0.001	<0.001	0.877
Age	61- (reference)						
	15–30	OR	1.413	0.795	0.741	1.700	1.118
		95%CI	1.307–1.529	0.737–0.858	0.674–0.814	1.449–1.995	0.835–1.496
		*p* Value	<0.001	<0.001	<0.001	<0.001	0.454
	31–45	OR	1.343	0.791	0.740	0.963	1.003
		95%CI	1.244–1.449	0.735–0.852	0.675–0.810	0.827–1.121	0.757–1.329
		*p* Value	<0.001	<0.001	<0.001	0.626	0.983
	46–60	OR	1.063	0.759	0.769	0.857	0.914
		95%CI	0.985–1.147	0.704–0.819	0.701–0.844	0.738–0.994	0.696–1.201
		*p* Value	0.117	<0.001	<0.001	0.042	0.521
Education	University/college (reference)						
	Illiterate	OR	0.572	0.766	0.878	0.755	0.604
		95%CI	0.623–0.625	0.695–0.844	0.777–0.993	0.615–0.927	0.416–0.977
		*p* Value	<0.001	<0.001	0.038	0.007	0.008
	Primary school	OR	0.698	0.913	0.914	0.815	0.783
		95%CI	0.659–0.739	0.865–0.963	0.852–0.981	0.705–0.944	0.598–1.025
		*p* Value	<0.001	0.001	0.012	0.006	0.075
	Junior middle school	OR	0.897	1.031	0.964	0.870	0.894
		95%CI	0.856–0.940	0.990–1.073	0.914–1.016	0.773–0.980	0.716–1.117
		*p* Value	<0.001	0.136	0.173	0.022	0.326
	Senior middle school	OR	1.118	1.113	1.008	0.892	1.114
		95%CI	1.064–1.175	1.068–1.160	0.955–1.065	0.788–1.010	0.875–1.418
		*p* Value	<0.001	<0.001	0.764	0.072	0.382
Marriage Status	Unmarried (reference)						
	Married	OR	0.971	1.288	1.327	2.560	1.098
		95%CI	0.931–1.013	1.238–1.340	1.257–1.401	2.268–2.891	0.869–1.387
		*p* Value	0.168	<0.001	<0.001	<0.001	0.432
Employment	Unemployed (reference)						
	Employed	OR	1.237	1.047	0.963	0.388	0.814
		95%CI	1.183–1.294	1.004–1.092	0.913–1.016	0.352–0.427	0.687–0.965
		*p* Value	<0.001	0.030	0.164	<0.001	0.018
Hukou status	Rural household registration (reference)						
	Urban account	OR	1.052	1.003	0.916	1.068	1.053
		95%CI	1.017–1.087	0.974–1.033	0.881–0.952	0.982–1.160	0.900–1.232
		*p* Value	0.003	0.837	<0.001	0.124	0.521
Range of migration	Intercounty (reference)						
	Intercity	OR	0.954	0.859	0.824	0.872	0.749
		95%CI	0.913–0.997	0.829–0.891	0.788–0.862	0.788–0.965	0.619–0.907
		*p* Value	0.036	<0.001	<0.001	0.008	0.003
	Interprovince	OR	0.653	0.549	0.487	0.655	0.610
		95%CI	0.627–0.680	0.530–0.569	0.465–0.509	0.593–0.723	0.506–0.735
		*p* Value	<0.001	<0.001	<0.001	<0.001	<0.001
Reasons for migration	Family (reference)						
	Work	OR	1.081	0.852	0.712	1.116	1.024
		95%CI	1.030–1.134	0.816–0.889	0.675–0.750	1.007–1.237	0.851–1.231
		*p* Value	0.002	<0.001	<0.001	0.036	0.804
	Others	OR	1.132	1.030	0.991	1.105	1.14
		95%CI	1.013–1.264	0.932–1.139	0.878–1.117	0.898–1.361	0.764–1.623
		*p* Value	0.028	0.858	0.877	0.347	0.575
Household income ranking	Highest (≥percentile 80)						
	Lowest (<percentile 20) (reference)	OR	0.957	1.108	1.219	1.064	1.014
		95%CI	0.912–1.005	1.060–1.159	1.148–1.295	0.933–1.213	0.794–1.294
		*p* Value	0.078	<0.001	<0.001	0.354	0.913
	Lower (percentile 20–39)	OR	1.050	1.072	1.108	1.050	0.931
		95%CI	1.000–1.103	1.025–1.120	1.044–1.177	0.919–1.199	0.728–1.192
		*p* Value	0.052	0.002	0.001	0.476	0.513
	Middle (percentile 40–59)	OR	1.080	1.066	1.108	1.023	0.961
		95%CI	1.029–1.134	1.020–1.114	1.045–1.175	0.896–1.169	0.747–1.236
		*p* Value	0.002	0.004	0.001	0.736	0.757
	Higher (percentile 60–79)	OR	1.135	1.062	1.104	1.036	1.032
		95%CI	1.082–1.192	1.017–1.108	1.041–1.170	0.907–1.182	0.797–1.336
		*p* Value	<0.001	0.006	0.001	0.605	0.811
Pseudo *R*^2^			0.024	0.043	0.055	0.151	0.026
*N*			135,372	108,668	110,274	15,302	4,423
Province of settlement			Control				

**Table 5:** Binary logistic regression results of the type of insurance coverage affecting the health service utilization behavior of the mobile population.

Variable			Public health service utilization behavior			Medical service utilization behavior	
			Health Education	Establishing a health record	Family doctor contract	Acceptance of hospitalization	Choose local hospitalization
Type of health insurance	BMISURR (reference)						
	BMIUE	OR	1.210	1.484	1.781	1.220	2.137
		95%CI	1.125–1.302	1.405–1.568	1.663–1.907	1.026–1.451	1.359–3.359
		p Value	<0.001	<0.001	<0.001	0.024	<0.001
Gender	Female (reference)						
	Male	OR	0.989	0.909	0.924	0.529	1.005
		95%CI	0.928–1.053	0.867–0.954	0.868–0.983	0.456–0.613	0.647–1.721
		*p* Value	0.721	<0.001	0.012	<0.001	0.831
Age	60 + (reference)						
	15–30	OR	1.377	0.679	0.598	1.347	1.060
		95%CI	1.111–1.707	0.566–0.815	0.489–0.732	0.934–1.942	0.118–9.557
		*p* Value	0.003	<0.001	<0.001	0.111	0.958
	31–45	OR	1.184	0.702	0.608	0.881	1.310
		95%CI	0.961–1.460	0.587–0.839	0.499–0.741	0.621–1.249	0.147–11.641
		*p* Value	0.113	<0.001	<0.001	0.477	0.809
	46–60	OR	0.977	0.734	0.690	0.799	1.006
		95%CI	0.791–1.206	0.612–0.879	0.3565–0.842	0.566–1.128	0.110–9.193
		*p* Value	0.826	<0.001	<0.001	0.202	0.996
Education	University/college (reference)						
	Illiterate	OR	0.612	0.730	0.830	0.536	1.039
		95%CI	0.481–0.780	0.587–0.908	0.642–1.073	0.340–0.846	0.247–4.375
		*p* Value	<0.001	0.005	0.155	0.007	0.958
	Primary school	OR	0.829	0.939	0.915	0.883	0.825
		95%CI	0.727–0.945	0.844–1.044	0.802–1.045	0.668–1.169	0.388–1.754
		*p* Value	0.005	0.243	0.190	0.386	0.617
	Junior middle school	OR	1.044	1.007	0.941	0.886	0.937
		95%CI	0.957–1.139	0.942–1.075	0.892–1.057	0.722–1.088	0.540–1.626
		*p* Value	0.337	0.846	0.497	0.248	0.817
	Senior middle school	OR	1.198	1.076	0.973	0.865	0.936
		95%CI	1.099–1.307	1.011–1.146	0.887–1.046	0.707–1.058	0.538–1.628
		*p* Value	<0.001	0.022	0.374	0.159	0.814
Marriage status	Unmarried (reference)						
	Married	OR	0.881	1.272	1.012	3.814	1.116
		95%CI	0.808–0.960	1.190–1.358	1.191–1.421	3.024–4.809	0.518–2.406
		*p* Value	0.004	<0.001	<0.001	<0.001	0.779
Employment	Unemployed (reference)						
	Employed	OR	1.402	0.983	1.025	0.426	0.799
		95%CI	1.265–1.555	0.904–1.069	0.915–1.119	0.351–0.517	0.512–1.246
		*p* Value	<0.001	0.688	0.818	<0.001	0.332
Hukou status	Rural household registration (reference)						
	Urban Account	OR	1.086	1.029	0.958	1.078	0.975
		95%CI	1.018–1.160	0.980–1.080	0.963–1.091	0.932–1.247	0.649–1.463
		*p* Value	0.013	0.254	0.433	0.310	0.902
Range of migration	Intercounty (reference)						
	Intercity	OR	0.967	0.865	0.855	0.853	0.400
		95%CI	0.870–1.074	0.806–0.928	0.786–0.931	0.692–1.051	0.166–0.964
		*p* Value	0.527	<0.001	<0.001	0.136	0.041
	Interprovince	OR	0.637	0.547	0.501	0.606	0.380
		95%CI	0.577–0.704	0.511–0.586	0.460–0.546	0.492–0.747	0.156–0.931
		*p* Value	<0.001	<0.001	<0.001	<0.001	0.034
Reasons for migration	Family (reference)						
	Work	OR	1.111	0.857	0.715	1.065	1.043
		95%CI	1.007–1.226	0.795–0.942	0.654–0.782	0.877–1.292	0.598–1.817
		*p* Value	0.035	<0.001	<0.001	0.527	0.882
	Others	OR	1.071	1.024	0.768	1.447	1.556
		95%CI	0.822–1.395	0.832–1.261	0.598–0.986	0.857–2.443	0.319–7.594
		*p* Value	0.610	0.820	0.038	0.167	0.585
Household income ranking	Highest (≥percentile 80) (reference)						
	Lowest (<percentile 20)	OR	1.139	1.085	1.180	0.973	0.885
		95%CI	1.025–1.266	0.999–1.178	1.062–1.312	0.762–1.241	0.446–1.757
		*p* Value	0.016	0.054	0.002	0.823	0.727
	Lower (percentile 20–39)	OR	1.289	1.091	1.124	1.049	0.704
		95%CI	1.164–1.426	1.010–1.177	1.017–1.242	0.830–1.326	0.373–1.330
		*p* Value	<0.001	0.026	0.022	0.688	0.279
	Middle (percentile 40–59)	OR	1.236	1.084	1.109	1.171	0.546
		95%CI	1.123–1.359	1.008–1.165	1.007–1.220	0.936–1.466	0.296–1.007
		*p* Value	<0.001	0.030	0.035	0.167	0.170
	Higher (percentile 60–79)	OR	1.272	1.087	1.183	1.056	0.053
		95%CI	1.163–1.391	1.016–1.164	1.081–1.294	0.851–1.310	0.437–1.551
		*p* Value	<0.001	0.016	<0.001	0.622	0.546
Pseudo *R*^2^			0.030	0.036	0.035	0.116	0.064
*N*			37,133	32,397	33,251	4,463	1,225
Province of settlement			Control				

BMISURR, Basic Medical Insurance System for Urban and Rural Residents; BMIUE, Basic Medical Insurance for Urban Employees.

Table 5 presents the regression results of the impact of health insurance types on the health service utilization behaviors of mobile populations. The results suggest that participating in urban employee medical insurance increases the likelihood of accessing public health services and medical care. Specifically, in health education (OR = 1.210, 95% CI = 1.125–1.302), health records (OR = 1.484, 95% CI = 1.405–1.568), and family doctor sign-up (OR = 1.781, 95% CI = 1.663–1.907) - three categories of public health service utilization behaviors, as well as whether hospitalization is involved (OR = 1.220, 95% CI = 1.026–1.451) and whether local hospitalization is received (OR = 2.137, 95% CI = 1.359–3.359) - two categories of medical service utilization behaviors, mobile populations with urban employee medical insurance are more likely to utilize these services compared to those participating in rural resident medical insurance. Furthermore, this difference is more pronounced in health care behaviors, as the OR values for the three health care behaviors are all greater than those for medical service behaviors.

### Analysis of the mediating mechanism of health insurance participation characteristics affecting health

#### Mediating mechanisms of participant location influencing health

**Figure 2:**
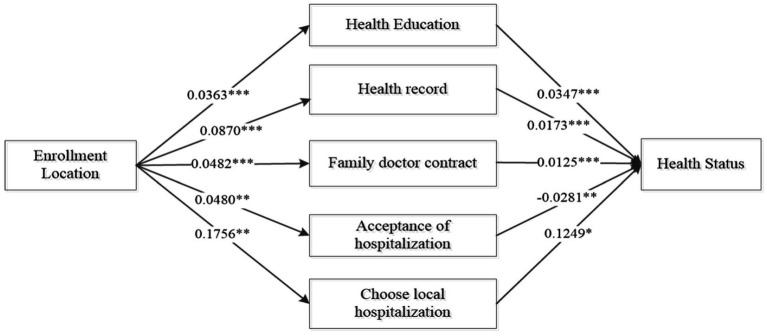
Pathway of the impact of the location of participation on the health status of the mobile Population. *indicates significant at the 10% level, **indicates significant at the 5% level, ***indicates significant at the 1% level.

Table 6 and Figure 2 illustrate the impact pathways of the location of participating in health insurance on the health of the mobile population. From the results, it can be observed that both health care behaviors and medical service behaviors play a partial mediating role in this impact pathway, indicating that whether to participate in local health insurance can influence the health of the mobile population through these channels. In terms of the main effect analysis, the main effect of whether to participate in local health insurance on the health of the mobile population is significant at the 1% statistical level. From the analysis of direct and indirect effects, whether in the intermediary variables of health education, health records, and family doctor signing in health care behaviors, or in the intermediary variables of whether hospitalization and local hospitalization in medical service utilization behaviors, their direct effects are all significant, and the confidence intervals of the indirect effects do not include 0, indicating that the indirect effects of these intermediary variables are also significant, thus indicating the existence of partial mediating effects. However, there are significant differences in the magnitude of the effects of different intermediary variables. Among them, the mediating effect of whether local hospitalization is the largest, with an effect size of 0.0214, followed by whether to establish health records locally, whether to receive health education, whether to be hospitalized, and whether to sign with a local family doctor, with mediating effect sizes of 0.0015, 0.0012, 0.0011, and 0.0006, respectively.

**Table 6:** Mediating mechanisms affecting the health of the mobile population at the location of health insurance participation.

Variable	Main effect	Intermediate variables	Direct effect	Indirect effects	upper limit	lower limit	Intermediary Effect	Intermediary Type
Enrollment location	−0.0207***	Health Education	−0.0219***	0.0013***	−0.0153	−0.0260	0.0012 ***	Partial intermediary role
		Establishing a health record	−0.0222***	0.0015***			0.0015***	Partial intermediary role
		Family doctor contract	−0.0213***	0.0006***			0.0006***	Partial intermediary role
	−0.0525***	Acceptance of hospitalization	−0.0514***	−0.0011***	−0.0301	−0.0749	−0.0011**	Partial intermediary role
	−0.0300***	Choose local hospitalization	−0.0514**	0.0214***	0.0111	−0.0712	0.0214***	Partial intermediary role

*indicates significant at the 10% level. **indicates significant at the 5% level. ***indicates significant at the 1% level.

#### Mediating mechanisms of health insurance types affecting the health of mobile populations

**Figure 3:**
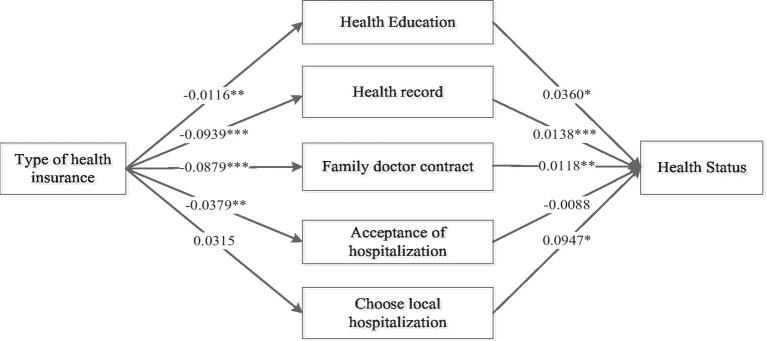
The pathway of the influence of the type of medical insurance on the health status of the mobile population. *indicates significant at the 10% level, **indicates significant at the 5% level, ***indicates significant at the 1% level.

Due to significant differences in payment ratios, coverage scope, and benefit levels between urban and rural residents’ medical insurance and urban employee medical insurance, there may be variations in the pathways through which these two insurances impact the health of migrant populations. Considering the advantages of urban employee medical insurance in terms of funding levels and reimbursement standards, and based on the above analysis results, it is evident that urban employee medical insurance has a more significant positive impact on the health of migrant populations. Therefore, this study takes urban residents’ medical insurance as a reference and focuses on analyzing the mediating mechanisms through which urban employee medical insurance influences the health of migrant populations, as shown in Table 7. From the results, it can be observed that in the analysis of the mediating role of urban employee medical insurance on the health of migrant populations, the main effects of urban employee medical insurance on the health of migrant populations are all significant. The direct effects and indirect effects of variables such as whether to receive health education, whether to establish a local health record, and whether to sign a contract with a local family doctor are all significant. This indicates that these three variables play a partial mediating role, with respective mediating effect sizes of 0.0004, 0.0012, and 0.0010. However, whether hospitalization and local hospitalization play a mediating role in the utilization of medical services behavior is not evident (Figure 3).

**Table 7:** Mediating mechanisms by which type of health insurance affects the health of mobile populations.

Variable	Main effect	Intermediate variables	Direct effect	Indirect effects	Upper limit	Lower limit	Intermediary effect	Intermediary type
Type of health insurance	0.0298***	Health Education	0.0302***	−0.0004**	0.0392	0.0203	−0.0004**	Partial intermediary role
		Establishing a health record	0.0311***	−0.0013***			−0.0012***	Partial intermediary role
		Family doctor contract	0.0308***	−0.0010**			−0.0010**	Partial intermediary role
	0.1303***	Acceptance of hospitalization	0.1305***	−0.0002	0.1725	0.0880	0.0003	Intermediary is not established
	0.1524***	Choose local hospitalization	0.1511***	0.0013	0.2270	0.0778	0.0013	Intermediary is not established

*indicates significant at the 10% level. **indicates significant at the 5% level. ***indicates significant at the 1% level.

## Discussion

In this study, we conducted an in-depth exploration of the impact and pathways of basic health insurance enrollment characteristics on the health of migrant populations, aiming to enhance the support of health insurance for the well-being of mobile populations. As the world’s most populous developing country with a significant migrant population, the empirical evidence from China holds substantial practical significance. The research findings indicate that health insurance exerts a positive influence on the utilization of healthcare services and the overall health of mobile populations. This conclusion aligns with previous studies on the relationship between health insurance and the health of migrant populations, such as research utilizing health survey data from Canada and the United States in 2002 and 2003. Through cross-national comparisons, researchers investigated the impact of health insurance on the disparities in access to primary healthcare services between immigrants and non-immigrants. They identified health insurance as a key factor contributing to these disparities and suggested the need to expand insurance coverage (50). However, compared to other countries, China’s mobile population faces more intricate challenges in health security. Firstly, the sheer number of China’s mobile population is larger and continues to grow rapidly. Secondly, China’s health insurance policies are more complex, with variations in policies between different regions (51). Additionally, regional disparities in healthcare resources further contribute to the unique nature of healthcare security for China’s mobile population. In order to strengthen health insurance coverage for the health security of mobile populations, the Chinese government has implemented various measures within the framework of healthcare system reform. These initiatives include providing free public health services for mobile populations through health insurance and establishing a nationwide unified reimbursement system (52). These efforts play a pivotal role in promoting the utilization of healthcare services and improving the health outcomes of China’s mobile population.

On the other hand, we need to pay attention to the differentiated impact of different health insurance enrollment characteristics on the utilization of healthcare services and health outcomes among the mobile population. This differentiation manifests in two main aspects. Firstly, individuals participating in local health insurance at their place of residence are more likely to access healthcare services and are also more likely to have better health statuses. Secondly, individuals enrolled in urban employee medical insurance are more likely to access healthcare services and tend to have better health conditions. Despite the integration of fragmented health insurance systems in China, significant policy disparities persist among different regions. These differences encompass levels of insurance coverage, coverage directories, and reimbursement procedures for medical expenses. Additionally, the lower-level coordination within the health insurance system restricts the transferability of health insurance relationships for the mobile population. Opting for local health insurance at the place of residence contributes to obtaining a more comprehensive level of coverage and helps avoid the complexities of reimbursement procedures and associated costs when seeking medical care in different locations. The reasons for the divergent impact of various types of health insurance systems may stem from their distinct targets within the Chinese population, resulting in differences across multiple facets of benefit levels (22). For instance, the mobile population, when covered by urban employee medical insurance compared to rural and urban resident medical insurance, enjoys higher reimbursement benefits, leading to more significant effects on health protection (53). According to the 2018 statistical report on the development of medical insurance released by the National Healthcare Security Administration of the People’s Republic of China, the reimbursement ratio for inpatient expenses within the scope of urban employee medical insurance policies is 81.6%, whereas it is 65.6% for rural and urban resident medical insurance policies (54). This implies a 16% difference in the reimbursement ratios between the two insurance systems. This form of unfairness in the utilization of health insurance is a prevalent issue globally. For example, a study utilizing nationwide reimbursement data from South Korea’s National Health Insurance between 2002 and 2010 evaluated the policy effects of expanding national health insurance coverage for cancer patients in 2005. The findings revealed that the policy partially alleviated income-related inequalities among inpatients in tertiary hospitals but did not improve income-related inequalities among outpatient cancer patients (55). Analyses of healthcare utilization inequalities in the United States also suggest that individuals with and without health insurance experience disparities in healthcare accessibility and overall health. Expanding health insurance coverage is more likely to enhance the quality of life and extend life expectancy (56).

Finally, the results of the intermediary mechanism analysis in this study indicate that health insurance not only has a direct positive impact on the health of the mobile population but also influences their health through the mediation of healthcare service utilization behaviors. In terms of the impact of the location of health insurance participation on the health of the mobile population, behaviors such as receiving health education, establishing health records, and signing contracts with local family doctors, as well as utilizing inpatient services and obtaining inpatient services locally, play a partial intermediary role. The most significant intermediary effect is observed in receiving inpatient services locally, possibly because the reimbursement focus in China’s health insurance reform is predominantly on inpatient services, providing comprehensive coverage for the mobile population’s hospitalization needs (57). In the pathway of the impact of health insurance types on the health of the mobile population, health education, health records, and family doctor signing behaviors play a partial intermediary role, while behaviors such as receiving inpatient services and obtaining inpatient services locally do not act as intermediaries in the mechanism. Therefore, to further enhance health coverage for the mobile population through health insurance, attention should be given to the indirect effects of healthcare service utilization behaviors in this influence.

Summing up the above analysis, this study has three main advantages compared to existing relevant research. Firstly, using large sample data, the study not only empirically verifies the impact of health insurance on the health of the mobile population but also delves deeper into the multidimensional analysis of the influence of health insurance on the utilization of healthcare services by the mobile population. This provides a crucial supplement to the current literature on improving the health and healthcare service utilization of the mobile population. Secondly, we examine the impact of different enrollment characteristics of health insurance on the health and service utilization of the mobile population. This holds practical significance for the government in enhancing the performance of healthcare security and reinforcing health protection for the mobile population. Thirdly, the study analyzes the intermediary role of healthcare service utilization, contributing to better health improvement for the mobile population and suggesting more effective health insurance policies to alleviate inequalities in healthcare security for the mobile population. However, the study has some limitations. Firstly, due to data constraints, we mainly focus on the intermediary role of two types of healthcare service behaviors, namely, public health service utilization and inpatient service utilization, in the relationship between health insurance and the health of the insured. Future research, with richer data, can further include other pathways such as individual health behaviors, outpatient service utilization, and medical expenses in intermediary mechanism analysis for a more comprehensive exploration of how health insurance affects the health of the mobile population. Secondly, the study utilizes cross-sectional data from the 2018 CMDS database, limiting our ability to determine trends or long-term associations between health insurance and the health of the mobile population. It also hinders the verification of specific causal relationships between mechanisms. In future research, adopting longitudinal or experimental designs could better determine the direction of causality between health insurance and health, ensuring more robust and reliable causal inferences. Thirdly, as the 2018 CMDS only uses self-assessed health status as the sole measurement criterion for the health condition of the mobile population, it becomes challenging to measure health status through other objective indicators. With richer data in the future, incorporating additional objective indicators to assess health could enhance the analysis.

## Policy implications

Based on our research findings, we offer some recommendations for reference. First, there is a need to further improve the accessibility of health services for the mobile population. In the future, more healthcare services could be included in the coverage of medical insurance to enhance the equality of public health services. The government can enhance disease and medical knowledge among the mobile population through health education and medical examinations, enabling them to scientifically assess their own health conditions and promote awareness of health management. The government should optimize the allocation of medical service resources, innovate service models, and improve the accessibility of medical services based on the characteristics of the mobile population’s work, residence, major health issues, and health conditions, ensuring that they can easily access medical services (21, 43). Second, there is a need to reduce the disparities in treatment between different regions and insurance systems. The government could further elevate the coordination level of basic medical insurance to promote the uniformity of medical insurance policies between different regions, facilitating the mobility and reimbursement of medical expenses for the mobile population. Regarding the differences in coverage between different medical insurance systems, efforts can be made to reduce disparities in aspects such as the deductible standard, payment ratio, maximum payment limit, and types of reimbursable diseases from the medical insurance fund to achieve equality in welfare benefits between different insurance systems (58). Third, simplifying the procedures for transferring medical insurance relationships is crucial. For mobile populations with longer durations of mobility, they could be encouraged to transfer their medical insurance relationships to their places of residence, actively encouraging participation in local medical insurance. Simultaneously, for mobile populations with formal employment, active encouragement to participate in urban employee medical insurance should be provided to enhance health security performance.

## Conclusion

The mobile population has made significant contributions to China’s economic and social development. With the continuous growth of the mobile population, addressing the health issues they face has become an integral part of building a Healthy China. This article, based on cross-sectional data from the 2018 CMDS database, analyzes the relationship between the characteristics of medical insurance enrollment and the health of the mobile population, as well as the mediating role of healthcare service utilization. The research results indicate that participating in local medical insurance and urban employee medical insurance significantly enhances the healthcare service utilization and overall health levels of the mobile population. At the same time, public health service utilization and hospital service utilization play important mediating roles in the relationship between medical insurance and the health of the mobile population. However, differences exist in the types of services regarding the nature of mediation and the size of mediating effects. Future efforts should focus on improving the accessibility of healthcare services for the mobile population, narrowing the disparities in treatment between different regions and insurance systems. This will further enhance the health security provided by medical insurance for the mobile population. These findings serve as a basis for refining policies related to the medical security of the mobile population and contribute to the realization of the goals of a Healthy China.

## Data availability statement

This study was based on a publicly available database, the China Migrants Dynamic Survey (CMDS), which was conducted annually by the National Health and Wellness Commission of China since 2009. The datasets generated and/or analyzed during the current study are available in the official website (https://www.chinaldrk.org.cn/).

## Ethics statement

Ethical approval was not required, as this study was a secondary analysis conducted using public data sets from the CMDS that did not include identifable personal information. Each volunteer participant obtained a written informed consent based on inclusion criteria. All procedures performed in this study were in accordance with the 1964 Helsinki declaration and its later amendments or comparable ethical standards.

## Author contributions

BD collected, cleaned and prepared the data, analyzed and interpreted the data, drafted the manuscript and made subsequent revisions, read and approved the final manuscript.
